# Current frailty knowledge, awareness, and practices among physicians following the 2022 European consensus document on Frailty in Cardiology

**DOI:** 10.1093/ehjopen/oeae025

**Published:** 2024-03-27

**Authors:** Jie Jun Wong, Laureen Yi-Ting Wang, Koji Hasegawa, Kay Woon Ho, Zijuan Huang, Louis L Y Teo, Jack Wei Chieh Tan, Kazuyuki Kasahara, Ru-San Tan, Junbo Ge, Angela S Koh

**Affiliations:** Department of Cardiology, National Heart Center Singapore, 5 Hospital Drive, 169609 Singapore, Singapore; Division of Cardiology, Alexandra Hospital, National University Health System, Singapore, Singapore; Division of Translational Research, National Hospital Organization Kyoto Medical Center, Kyoto, Japan; Department of Cardiology, National Heart Center Singapore, 5 Hospital Drive, 169609 Singapore, Singapore; Duke-NUS Medical School, 8 College Road, 169857 Singapore, Singapore; Department of Cardiology, National Heart Center Singapore, 5 Hospital Drive, 169609 Singapore, Singapore; Duke-NUS Medical School, 8 College Road, 169857 Singapore, Singapore; Department of Cardiology, National Heart Center Singapore, 5 Hospital Drive, 169609 Singapore, Singapore; Duke-NUS Medical School, 8 College Road, 169857 Singapore, Singapore; Department of Cardiology, National Heart Center Singapore, 5 Hospital Drive, 169609 Singapore, Singapore; Duke-NUS Medical School, 8 College Road, 169857 Singapore, Singapore; Lee Kong Chian School of Medicine, Nanyang Technological University, Singapore, Singapore; Department of Cardiology, National Heart Center Singapore, 5 Hospital Drive, 169609 Singapore, Singapore; Duke-NUS Medical School, 8 College Road, 169857 Singapore, Singapore; Department of Cardiology of Zhongshan Hospital, Fudan University, Shanghai, China; Department of Cardiology, National Heart Center Singapore, 5 Hospital Drive, 169609 Singapore, Singapore; Duke-NUS Medical School, 8 College Road, 169857 Singapore, Singapore

**Keywords:** Frailty, Cardiologists, Knowledge, Awareness, Practices

## Abstract

**Aims:**

Aging-related cardiovascular disease and frailty burdens are anticipated to rise with global aging. In response to directions from major cardiovascular societies, we investigated frailty knowledge, awareness, and practices among cardiologists as key stakeholders in this emerging paradigm a year after the European Frailty in Cardiology consensus document was published.

**Methods and results:**

We launched a prospective multinational web-based survey via social networks to broad cardiology communities representing multiple World Health Organization regions, including Western Pacific and Southeast Asia regions. Overall, 578 respondents [38.2% female; ages 35–49 years (55.2%) and 50–64 years (34.4%)] across subspecialties, including interventionists (43.3%), general cardiologists (30.6%), and heart failure specialists (HFSs) (10.9%), were surveyed. Nearly half had read the consensus document (38.9%). Non-interventionists had better perceived knowledge of frailty assessment instruments (fully or vaguely aware, 57.2% vs. 45%, adj. *P* = 0.0002), exercise programmes (well aware, 12.9% vs. 6.0%, adj. *P* = 0.001), and engaged more in multidisciplinary team care (frequently or occasionally, 52.6% vs. 41%, adj. *P* = 0.002) than interventionists. Heart failure specialists more often addressed pre-procedural frailty (frequently or occasionally, 43.5% vs. 28.2%, *P* = 0.004) and polypharmacy (frequently or occasionally, 85.5% vs. 71%, adj. *P* = 0.014) and had consistently better composite knowledge (39.3% vs. 21.6%, adj. *P* = 0.001) and practice responses (21% vs. 11.1%, adj. *P* = 0.018) than non-HFSs. Respondents with better knowledge responses also had better frailty practices (40.3% vs. 3.6%, adj. *P* < 0.001).

**Conclusion:**

Distinct response differences suggest that future strategies strengthening frailty principles should address practices peculiar to subspecialties, such as pre-procedural frailty strategies for interventionists and rehabilitation interventions for HFSs.

## Graphical Abstract

**Graphical Abstract:**
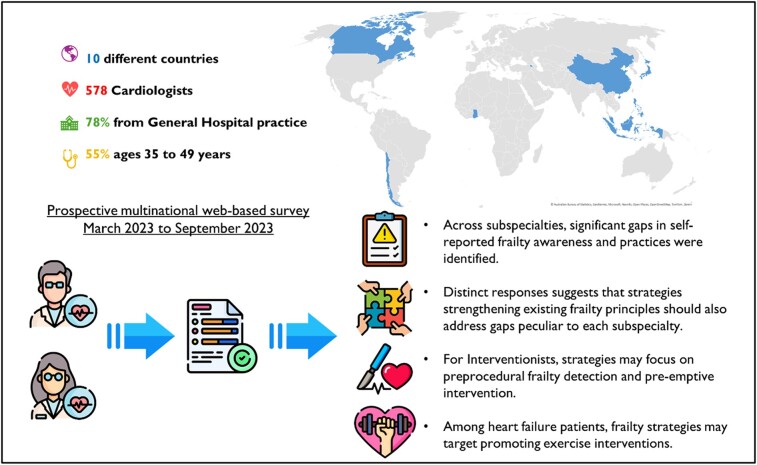
The icons were made by *Smashicons*, *Nhor Phai*, and *Freepik* at www.flaticon.com. Microsoft product screenshot(s) reprinted with permission from Microsoft Corporation.

**Lay summary**

- Patients with cardiovascular disease are at higher risk of developing frailty and complications, with cardiovascular health being a central tenet of frailty. With a global aging population, aging-related cardiovascular disease and frailty are concurrently anticipated to rise. In response, we launched a multinational survey to broad cardiology communities worldwide to understand the frailty knowledge, awareness, and practices among cardiologists across various subspecialties.
- Of 578 cardiologists originating from 10 different countries and 10 different subspecialties surveyed, most respondents had low self-perceived frailty awareness and knowledge, but the majority had recognized the importance of frailty and frailty intervention.
- Distinct response differences between subspecialties were identified, suggesting that future frailty intervention strategies should address frailty practices peculiar to each subspecialty, such as pre-procedural strategies for interventionists and rehabilitation interventions for heart failure specialists.

## Introduction

The cardiovascular system plays a central role in the pathogenesis of frailty.^1^ Cardiovascular diseases (CVDs) increase the risk of developing frailty, and frailty increases the cardiovascular mortality risk.^2^ Few studies, however, have prioritized physicians as key stakeholders for tackling frailty in CVD.

In 2022, the European Society of Cardiology (ESC) published its first consensus document on *Frailty in Cardiology*, detailing gaps in practices, the need for strategies for specific CVD, and multidisciplinary team-based care while emphasizing the importance of preoperative frailty assessments before cardiac procedures and a multidimensional approach to addressing coexistent multimorbidity in heart failure (HF).^3,4^ Frailty was recognized as heterogeneous and population specific^4^; therefore, a comprehensive, unselected approach may disproportionately consume resources for assessments rather than the actual intervention implementation.^5^ Identifying specific patient and physician requirements may instead enable a more effective, sustainable, longer-term implementation.^5^

One year after the ESC consensus was published, we describe the current frailty knowledge, awareness, and practices across various cardiology subspecialties. We hypothesize that each subspecialty provides different perceptions regarding frailty management in CVD necessary for overcoming future challenges related to implementation of frailty interventions.

## Methods

This investigator-initiated survey was initially designed in English and later translated into Mandarin and Japanese by native language-speaking co-authors and published separately on SurveyMonkey (Momentive, Waterford, NY, USA). The final survey comprised 31 questions and was accessible for 7 months, from March 2023 to September 2023. Two successive invitations were sent.

Pre-specified subgroup analysis was performed for self-declared primary subspecialties. Multivariable logistic regression was adjusted for significant baseline characteristics. All *P*-values were two tailed. Statistical analyses were performed with IBM SPSS Statistics, Version 29 (IBM Corp, Armonk, NY, USA).

## Results

### Baseline characteristics

Nineteen non-cardiologist respondents were excluded, including administrators and allied health professionals. Of 578 cardiologist respondents (women, 38.2%), most were ages 35–64 and subspecialized in coronary intervention, general cardiology, and HF (*Table 1*). Most practiced in Asia–Pacific countries. Coronary and structural interventionists were analysed as ‘interventionists’.

**Table 1:** Self-reported characteristics of study participants

Baseline characteristics	Overall, *n* = 578
Age	
21–34	55 (9.5%)
35–49	319 (55.2%)
50–64	199 (34.4%)
65 and above	5 (0.9%)
Sex	
Female	221 (38.2%)
Male	357 (61.8%)
Country of work	
Armenia	1 (0.2%)
Canada	1 (0.2%)
Chile	2 (0.3%)
China	474 (82%)
Ghana	1 (0.2%)
Indonesia	2 (0.3%)
Japan	30 (5.2%)
Malaysia	4 (0.7%)
Philippines	4 (0.7%)
Singapore	56 (9.7%)
Practice setting	
General hospital	451 (78%)
Private hospital	41 (7.1%)
Private practice (non-hospital setting)	4 (0.7%)
University hospital	74 (12.8%)
Others	7 (1.2%)
Primary subspecialty in cardiology^a^	
Cardiac imaging	22 (3.8%)
Cardiac rehabilitation	12 (2.1%)
Critical care cardiology	18 (3.1%)
Electrophysiology	25 (4.3%)
General cardiology	177 (30.6%)
Heart failure	63 (10.9%)
Interventional (coronary)	245 (42.4%)
Interventional (structural heart disease)	5 (0.9%)
Pulmonary hypertension	2 (0.3%)
Women’s health/cardio-obstetrics	1 (0.2%)
Fellow or resident	8 (1.4%)
What are the types of cardiac conditions you manage most of the time?^b^	
Ischaemic heart disease/acute myocardial infarction/CAD	543 (93.9%)
Adult congenital or congenital heart disease	46 (8%)
Atrial fibrillation	396 (68.5%)
Other arrhythmias	155 (26.8%)
Heart failure	483 (83.6%)
Inherited or acquired cardiomyopathies	35 (6.1%)
Structural and valvular heart disease	107 (18.5%)
Interventional cardiology	229 (39.6%)
Cardiac imaging	57 (9.9%)
Cardio-oncology	8 (1.4%)
Pulmonary hypertension	49 (8.5%)
Cardiovascular critical care	110 (19%)
Preventive cardiology	103 (17.8%)
Women’s health/cardio-obstetrics	16 (2.8%)
Paediatric cardiology	1 (0.2%)
Other	7 (1.2%)

CAD, coronary artery disease.

^a^Respondents were allowed to select only one option.

^b^Respondents were allowed to select more than one option.

### Overall responses

**Figure 1:**
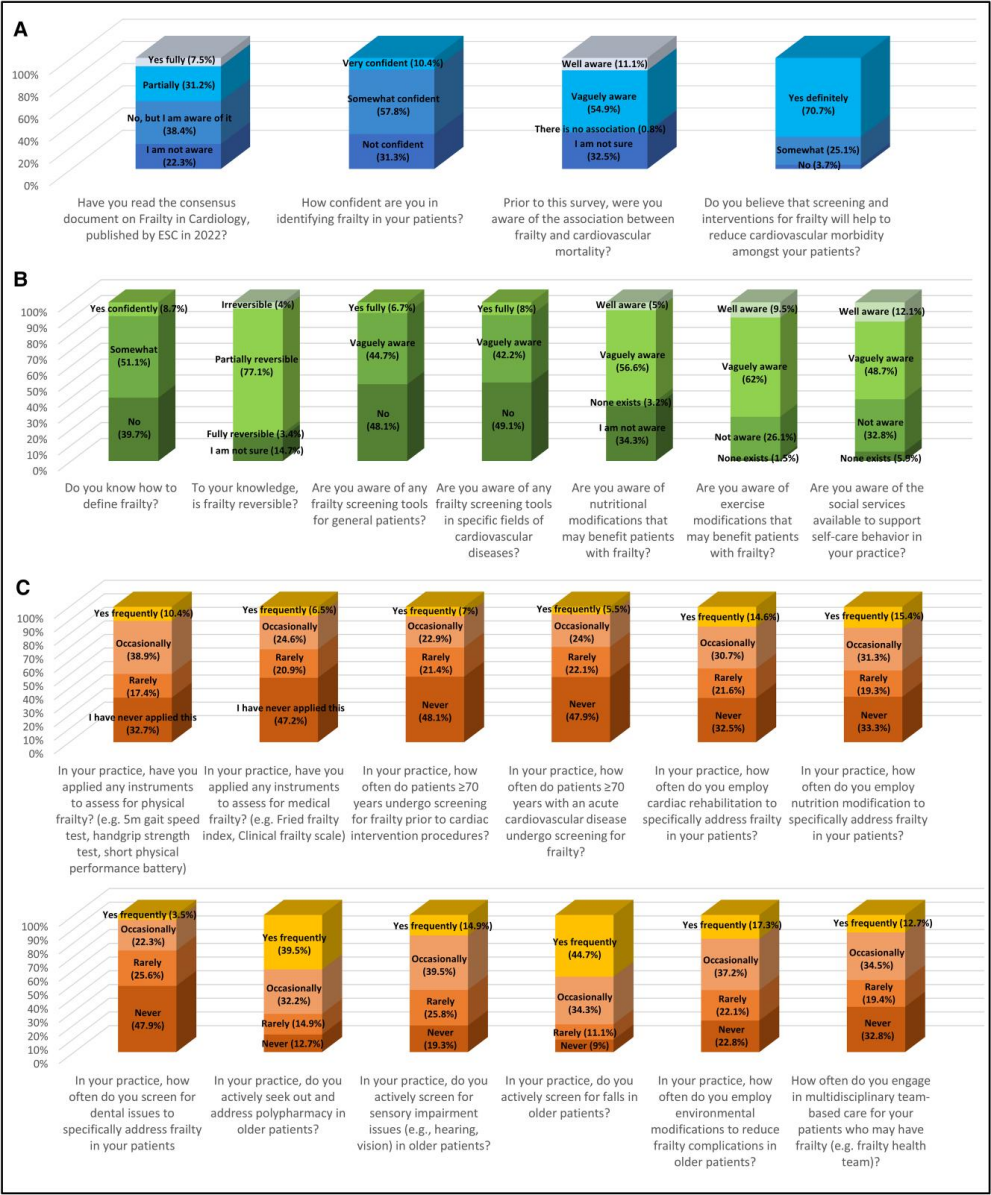
Self-reported frailty awareness, knowledge, and practices of overall respondents. Survey responses are grouped into awareness (*A*, first row), knowledge (*B*, second row), and practices (*C*, third and fourth rows). Data labels represent response options (% of respondents). ESC, European Society of Cardiology.

Approximately one-third of respondents were unsure how to define frailty (39.6%), unaware of the associations between frailty and cardiovascular mortality (32.5%), and not confident in identifying frailty (30.4%; *Figure 1*). Half were unaware of any screening tools for general frailty (47.9%) or specific CVD (49.3%). One-third were unaware of social services to support self-care (32.7%), and one-quarter were unaware of frailty exercise interventions (26.5%). Most respondents did not employ cardiac rehabilitation (never/rarely, 54.6%), nutritional measures (never/rarely, 53.1%), or engage in multidisciplinary care (never/rarely, 52.3%) to address frailty. Within their practices, most patients ≥70 years old did not receive frailty screening before cardiac procedures (never/rarely, 69.9%) or during acute CVD (never/rarely, 69.9%). Almost half of the respondents had read the ESC consensus document (fully/partially, 38.9%).

However, most respondents screened older patients for falls (frequently/occasionally, 79.6%), hearing or vision impairment (frequently/occasionally, 54.2%), and polypharmacy (frequently/occasionally, 72.3%; *Figure 1*). Almost all believed that frailty screening and interventions would reduce morbidity (believed/somewhat, 96.3%). Using composite responses, respondents with consistently better awareness and knowledge responses (fully/somewhat aware) also had consistently better frailty practices (frequently/occasionally, 40.3% vs. 3.6%, *P* < 0.001).

### Responses by interventionists

Among interventionists, over one-third were unaware of the associations between frailty and mortality, and half were unaware of any frailty screening tools (*Table 2*). In their practices, nearly three-quarters of older patients did not receive frailty screening before cardiac procedures or during acute CVD. Almost two-thirds of interventionists did not utilize rehabilitation, nutritional measures, or engage in multidisciplinary care. Conversely, non-interventionists had better awareness of frailty tools and social services and were more likely to utilize rehabilitation, nutritional modification, and multidisciplinary care than interventionists after adjusting for sex differences.

**Table 2:** Self-reported frailty awareness, knowledge, and practices, according to interventional subspecialty

	Non-interventional, *n* = 328	Interventional, *n* = 250	*P*-value	Adj. *P*-value^c^
Awareness				
1. Have you read the consensus document on *Frailty in Cardiology*, published by ESC in 2022?				
Not read or unaware	217 (66.4%)	133 (53.6%)	0.002	0.008
Read, fully or partially	110 (33.6%)	115 (46.4%)		
2. How confident are you in identifying frailty in your patients?				
Not confident	105 (32.1%)	71 (28.5%)	0.353	
Very or somewhat confident	222 (67.9%)	178 (71.5%)		
3. Prior to this survey, were you aware of the association between frailty and CV mortality?				
No association or unsure	96 (29.4%)	96 (38.7%)	0.019	0.011
Well or vaguely aware	231 (70.6%)	152 (61.3%)		
4. Are you aware of any frailty screening tools for general patients?				
Unaware	140 (42.8%)	137 (55%)	0.004	0.002
Fully or vaguely aware	187 (57.2%)	112 (45%)		
5. Are you aware of any frailty screening tools in specific CVD fields? (e.g. arrhythmias, valve disease, HF, PAD)				
Unaware	150 (45.9%)	135 (54.4%)	0.042	0.045
Fully or vaguely aware	177 (54.1%)	113 (45.6%)		
6. Do you believe frailty screening and interventions will help reduce CV morbidity amongst your patients?				
No	9 (2.8%)	10 (4%)	0.400	
Yes, definitely, or somewhat	318 (97.2%)	239 (96%)		
Knowledge				
7. Do you know how to define frailty?				
Not confident	129 (39.4%)	100 (40.2%)	0.863	
Confidently or somewhat	198 (60.6%)	149 (59.8%)		
8. To your knowledge, is frailty reversible?				
Fully or non-reversible, or unsure	68 (20.9%)	57 (22.9%)	0.571	
Partially reversible	257 (79.1%)	192 (77.1%)		
9. Are you aware of nutritional modifications that may benefit patients with frailty?				
None exists or unaware	112 (34.4%)	106 (42.7%)	0.040	0.015
Well or vaguely aware	214 (65.6%)	142 (57.3%)		
10. Are you aware of exercise modifications that may benefit patients with frailty?				
Vaguely or not aware, or non-existent	283 (87.1%)	234 (94%)	0.006	0.001
Well aware	42 (12.9%)	15 (6%)		
11. Are you aware of the social services available to support self-care behaviour in your practice?				
None exists or unaware	113 (34.6%)	110 (44.2%)	0.019	0.004
Well or vaguely aware	214 (65.4%)	139 (55.8%)		
Practices				
12. In your practice, have you applied any instruments to assess for physical frailty? (e.g. 5 m gait speed test, handgrip strength test, short physical performance battery				
Rarely or never	158 (48.5%)	133 (53.4%)	0.240	
Frequently or occasionally	168 (51.5%)	116 (46.6%)		
13. In your practice, have you applied any instruments to assess for medical frailty? (e.g. Fried’s index, CFS)				
Rarely or never	214 (65.6%)	181 (72.7%)	0.071	
Frequently or occasionally	112 (34.4%)	68 (27.3%)		
14. In your practice, how often do patients ≥70 years undergo screening for frailty prior to cardiac intervention procedures (e.g. pacemaker implantation, transcatheter aortic valve implantation, PCI, PAD)?				
Rarely or never	223 (68.2%)	181 (72.7%)	0.243	
Frequently or occasionally	104 (31.8%)	68 (27.3%)		
15. In your practice, how often do patients ≥70 years with an acute CVD undergo screening for frailty?				
Rarely or never	225 (68.8%)	181 (72.7%)	0.311	
Frequently or occasionally	102 (31.2%)	68 (27.3%)		
16. In your practice, how often do you employ cardiac rehabilitation to specifically address frailty?				
Rarely or never	167 (51.1%)	149 (60.1%)	0.031	0.010
Frequently or occasionally	160 (48.9%)	99 (39.9%)		
17. In your practice, how often do you employ nutrition modification to specifically address frailty?				
Rarely or never	156 (47.9%)	151 (60.6%)	0.002	<0.001
Frequently or occasionally	170 (52.1%)	98 (39.4%)		
18. In your practice, how often do you screen for dental issues to specifically address frailty in your patients?				
Rarely or never	236 (72.4%)	192 (77.1%)	0.199	
Frequently or occasionally	90 (27.6%)	57 (22.9%)		
19. In your practice, do you actively seek out and address polypharmacy in older patients?				
Rarely or never	81 (24.8%)	77 (30.9%)	0.101	
Frequently or occasionally	246 (75.2%)	172 (69.1%)		
20. How often do you actively screen for sensory impairment issues (e.g. hearing, vision) in older patients?				
Rarely or never	144 (44%)	119 (47.8%)	0.370	
Frequently or occasionally	183 (56%)	130 (52.2%)		
21. In your practice, do you actively screen for falls in older patients?				
Rarely or never	59 (18.1%)	55 (22.2%)	0.225	
Frequently or occasionally	267 (81.9%)	193 (77.8%)		
22. How often do you employ environmental modifications to reduce frailty complications in older patients?				
Rarely or never	136 (41.7%)	124 (49.8%)	0.054	
Frequently or occasionally	190 (58.3%)	125 (50.2%)		
23. How often do you engage in multidisciplinary team care for your patients who may have frailty? (e.g. Frailty team)				
Rarely or never	155 (47.4%)	147 (59%)	0.006	0.002
Frequently or occasionally	172 (52.6%)	102 (41%)		
Composite responses				
Awareness and knowledge responses^a^				
Unaware	240 (73.8%)	199 (79.9%)	0.089	
Fully aware, or somewhat	85 (26.2%)	50 (20.1%)		
Practice responses^b^				
Rarely or never	284 (87.1%)	221 (88.8%)	0.552	
Frequently or occasionally	42 (12.9%)	28 (11.2%)		

Comparisons were made using *χ*^2^ tests. *P*-values are two tailed.

CVD, cardiovascular disease; CFS, Clinical Frailty Scale; HF, heart failure; PAD, peripheral artery disease.

^a^Awareness and knowledge composite responses were tabulated from questions 2–5 and 6–11.

^b^Practice composite responses were tabulated from questions 12–23.

^c^Adjusted for sex.

### Responses by heart failure specialists

Heart failure specialists (HFSs) had better knowledge of exercise therapies (well aware, 18% vs. 9%, adj. *P* = 0.008), nutrition measures (well/vaguely aware, 77.0% vs. 60.2%, adj. *P* = 0.007), and social services (well aware, 24.2% vs. 11.1%, adj. *P* = 0.002) than non-HFS. Heart failure specialists were more likely to screen older patients before cardiac procedures (frequently/occasionally, 43.5% vs. 28.2%, adj. *P* = 0.004), address polypharmacy (frequently/occasionally, 85.5% vs. 71.0%, adj. *P* = 0.0014), and participate in multidisciplinary care (frequently, 22.6% vs. 11.9%, adj. *P* = 0.011). Heart failure specialists responded consistently better for awareness and knowledge questions (fully/somewhat aware, 39.3% vs. 21.6%, adj. *P* = 0.001) and had better frailty practices (frequently/occasionally, 21.0% vs. 11.1%, adj. *P* = 0.018).

However, over one-quarter of HFSs were not confident in identifying frailty (25.8%), and one-third were unaware of any tools for general frailty screening (38.7%) or specific CVD (38.7%). Most did not perform screening during acute CVD (rarely/never, 61.3%) or employ rehabilitation (rarely/never, 43.5%) or nutritional measures (rarely/never, 41.9%) against frailty.

## Discussion

**Figure 2:**
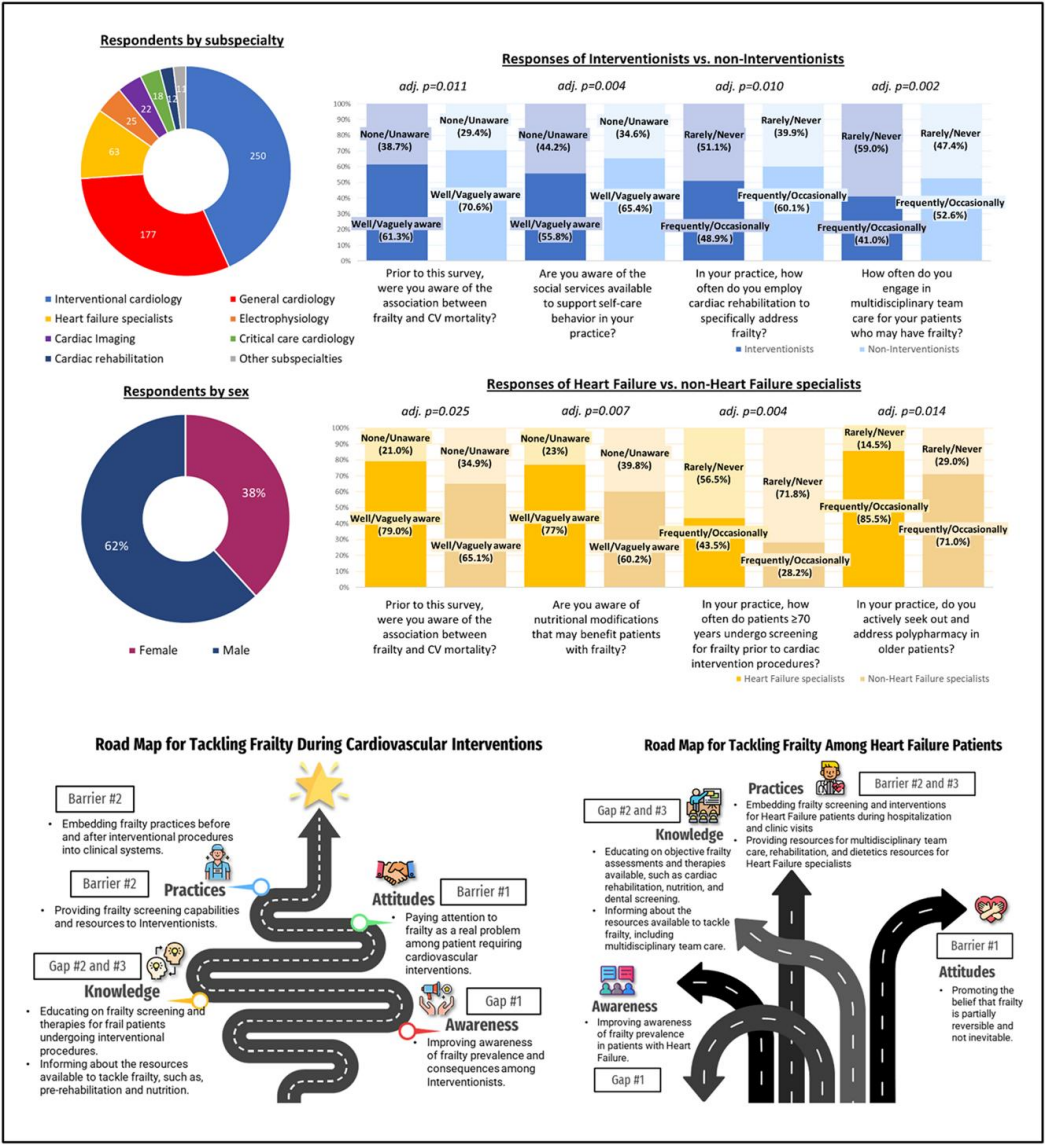
Frailty perceptions and suggested road maps for strategies tackling frailty across cardiology subspecialties. The recent European consensus document on *Frailty in Cardiology* recommends a validated, comprehensive approach to frailty model at pre- and post-acute cardiovascular event time points that leverages on a structured multidisciplinary care. The document recommends identifying specific needs from the individual’s frailty component to tailor intervention and recognizes cardiac rehabilitation, nutrition, and exercise as therapies that may reverse the degree of frailty. We surveyed cardiologists worldwide and found the majority of respondents had low self-perceived frailty awareness and knowledge, but most had recognized the importance of frailty and frailty intervention. Most respondents never or rarely used validated frailty assessment tools, and more than half never or rarely utilized frailty screening during acute illness or before cardiac procedures. Distinct response differences between subspecialties were identified. Non-interventionists had better frailty knowledge and were more likely to employ frailty strategies, such as cardiac rehabilitation, nutritional modification, and multidisciplinary care than interventionists. Heart failure specialists had better frailty knowledge than non-heart failure specialists and were more likely to screen older patients pre-procedurally, address frailty issues, including polypharmacy and dental problems, and engage in multidisciplinary care. Respondents with better knowledge responses also had better frailty practices. Gaps and barriers to the successful implementation of frailty services were identified for patients undergoing cardiovascular intervention procedures and patients with heart failure. Distinct response patterns indicating unique shortfalls and needs peculiar to each subspecialty were identified. Strategies targeting specific frailty needs between subspecialties are more likely to achieve effective and sustainable implementation and are, therefore, crucial for future frailty implementation research within cardiology. Icons were made by *Flat Icons*, *Eucalyp*, and *Freepik* at www.flaticon.com. Road map templates were made by *Slidesgo* and *Freepik* at www.slidesgo.com. Microsoft product screenshot(s) reprinted with permission from Microsoft Corporation.

To our knowledge, this is the first survey of frailty knowledge, awareness, and practices among cardiologists in the available literature. In this multinational survey, our findings highlight current gaps in frailty implementation and suggest that strategies should identify and address issues peculiar to each cardiology subspecialty (*Figure 2*).

Poor self-reported frailty awareness and practices were identified among cardiologists. Most respondents were not confident identifying frailty and did not screen for or utilize frailty interventions. However, most still believed in adequately tackling frailty, suggesting gaps between their beliefs and actual practices. These value–action gaps represent a practical, implementable target for improving frailty interventions within cardiology. Other studies not specific to cardiology have identified various barriers to frailty implementations, including a lack of dedicated resources, training opportunities, acceptance from hospital managers, and consensus on the appropriate frailty assessments and follow-up management, compounded by lack of understanding of the optimal method and timing of frailty interventions.^6,7^ While these factors apply generally, our findings suggest that there may be additional issues unique to cardiology settings, such as frailty screening before procedures or during acute CVD.

Interestingly, distinct patterns of frailty responses were observed between cardiology subspecialties. These differences in subspecialties’ requirements are novel findings that might add a layer of complexity to frailty strategies in cardiology.^8^ Surprisingly, interventionists reported low utilization of pre-procedural frailty screening, rehabilitative measures, and multidisciplinary care despite the high prevalence of older patients requiring cardiac procedures and the association with adverse procedural outcomes.^2^ This is despite data supporting periprocedural rehabilitation and nutritional measures among frail patients.^9^ Investment of resources towards frail CVD patients receiving cardiac procedures in the interventional laboratories may be necessary.^10^ Deliberate and targeted resource allocation that are additionally adaptable to patient-specific frailty may produce more effective and sustainable implementations (Graphic Abstract).^5^ Therefore, identifying the particular needs of cardiology subspecialties may be essential for future frailty implementation research on top of CVD disease-specific recommendations [see Supplement (Annex 1) of the ESC *Frailty in Cardiology* consensus document].^3^

Although HFS had better responses than non-HFS, significant gaps still need to be addressed. Heart failure patients often have considerable multimorbidity and high frailty prevalence strongly associated with adverse outcomes, including increased mortality and HF readmissions.^11^ Therefore, frailty is a pertinent issue among HF patients that needs to be promptly identified and addressed. However, many HFS respondents were unfamiliar with general frailty screening tools or tools for specific CVD and were not confident in identifying frailty. Many did not screen for frailty during acute CVD among older patients or utilize rehabilitative or nutritional interventions to tackle frailty. More efforts are still needed to improve frailty practices, even among HFS, even though they had better self-reported frailty scores.

Encouragingly, respondents with consistently better frailty awareness and knowledge responses also had better screening and intervention practices. This suggests that dissemination of frailty awareness with educational materials such as the ESC Frailty in Cardiology consensus document could translate into better practices.^12^ In addition, given the vast demographic and cultural differences in cardiology practices across the globe, broad-based and multiprong approaches transmitting these resources through various channels may be necessary to tackle frailty at a global scale.^13^

### Limitations

We acknowledge several limitations. As a self-reported survey, the results reflect respondents’ perceptions rather than actual knowledge or practices. Report biases inherent to surveys are possible. It is also impossible to verify whether responses reflect actual clinical practice. While it is possible that respondents might have over-reported favourable responses, our results continue to demonstrate prominent gaps in current frailty practices. As an open access survey, despite best efforts to have dedicated mailing lists, misclassification of respondents (as cardiologists) could still occur. However, our findings are consistent with other non-CVD studies reporting gaps in physician-level frailty practices; hence, we believe the results reflect responses from physicians.^6,14^ Finally, most respondents were from Asian countries, which limits the study’s generalizability to non-Asian practices. However, while the prevalence of frailty in Asia is higher than the average global frailty rate for now, our findings are critical not only for aging Asian societies but also for other global communities that will eventually grapple with aging populations.^15^

## Conclusions

Although most cardiologists recognized the importance of frailty screening and intervention, significant gaps in self-reported frailty awareness and practices were identified. We observed distinct patterns of responses among various cardiology subspecialties, indicating unique subspeciality shortfalls and needs, underscoring the importance of an individualized frailty approach for each subspecialty practice.

## Data Availability

The article’s data cannot be shared publicly due to institutional restrictions. The data will be shared upon reasonable request to the corresponding author.

## Lead author biography

Dr Jie Jun Wong is a cardiology fellow who is passionate about being a physician–scientist. His research interests include cardiovascular intervention, population health, prevention, implementation research, cardiovascular aging, frailty, and sarcopaenia. Regardless of socioeconomic standing, global populations inevitably will soon concurrently confront aging-related cardiovascular disease and frailty in the next decade, which will exacerbate existing healthcare system burdens. Understanding the practical barriers and challenges in the implementation of frailty strategies within resource limitations is a critical step in ensuring that our approaches to frailty interventions are effective, sustainable, and meaningful for our patients.
